# A stochastic T cell response criterion

**DOI:** 10.1098/rsif.2012.0205

**Published:** 2012-06-28

**Authors:** James Currie, Mario Castro, Grant Lythe, Ed Palmer, Carmen Molina-París

**Affiliations:** 1 Department of Applied Mathematics, School of Mathematics, University of Leeds, Leeds LS2 9JT, UK; 2 Grupo de Dinámica No-Lineal and Grupo Interdisciplinar de Sistemas Complejos (GISC) and Escuela Técnica Superior de Ingeniería (ICAI), Universidad Pontificia Comillas, 28015, Madrid, Spain; 3 Departments of Biomedicine and Nephrology, University Hospital, 4031 Basel, Switzerland

## Abstract

The adaptive immune system relies on different cell types to provide fast and coordinated responses, characterized by recognition of pathogenic challenge, extensive cellular proliferation and differentiation, as well as death. T cells are a subset of the adaptive immune cellular pool that recognize immunogenic peptides expressed on the surface of antigen-presenting cells by means of specialized receptors on their membrane. T cell receptor binding to ligand determines T cell responses at different times and locations during the life of a T cell. Current experimental evidence provides support to the following: (i) sufficiently long receptor–ligand engagements are required to initiate the T cell signalling cascade that results in productive signal transduction and (ii) counting devices are at work in T cells to allow signal accumulation, decoding and translation into biological responses. In the light of these results, we explore, with mathematical models, the timescales associated with T cell responses. We consider two different criteria: a stochastic one (the mean time it takes to have had *N* receptor–ligand complexes bound for at least a dwell time, *τ*, each) and one based on equilibrium (the time to reach a threshold number *N* of receptor–ligand complexes). We have applied mathematical models to previous experiments in the context of thymic negative selection and to recent two-dimensional experiments. Our results indicate that the stochastic criterion provides support to the thymic affinity threshold hypothesis, whereas the equilibrium one does not, and agrees with the ligand hierarchy experimentally established for thymic negative selection.

## 1. Summary

The binding properties of T cell receptors for self-pMHC (peptide–major histocompatibility complex) ligands are the basis for the selection in the thymus of a useful (MHC-restricted) and safe (self-tolerant) T cell repertoire. There exists a wealth of experimental support for the following: (i) T cell receptors must be bound to their ligands for a sufficiently long time to initiate the T cell signalling cascade and (ii) T cells require T cell receptor signal accumulation, which will be translated into appropriate biological responses. We have made use of mathematical models to test two different hypotheses: (a) the timescale of a T cell response correlates with the time it takes to have had *N* receptor–ligand complexes bound for at least a threshold dwell time, *τ*, each and (b) the timescale of a T cell response correlates with the time a threshold number, *N*, of TCRs must be occupied at equilibrium. We demonstrate that scenario (a) provides, for a given T cell receptor, a ligand hierarchy that agrees with that experimentally established for thymic negative selection, and an intuitive way to understand self–non-self discrimination of pMHC ligand. Our results suggest that a very small number (fewer than 10) of cognate ligand molecules is sufficient to elicit a T cell response, which is consistent with the serial engagement model.

## 2. Introduction

The adaptive immune system relies on different cell types to provide fast and coordinated responses, characterized by recognition of the pathogenic challenge, extensive cellular proliferation (division) and differentiation, as well as cellular death. T cells are a subset of the adaptive immune cellular pool that recognize immunogenic peptides (non-covalently bound to MHC class I and class II molecules expressed on the surface of antigen-presenting cells (APCs)) by means of specialized receptor molecules on their membrane. A (human or mouse) T cell expresses about 30 000 copies [1] of a T cell receptor molecule (TCR), whose ligands (usually referred to as antigens, in this context) are complexes composed of a peptide bound to an MHC molecule (pMHC). T cell receptors are both degenerate (a given TCR can recognize different pMHC complexes) and specific (single or point mutations to a pMHC complex can significantly alter recognition); yet TCR–pMHC interactions have low affinities [2–6]. *In vivo*, pMHC complexes are expressed on the surface of APCs; each (human or mouse) APC displays around 100 000 different pMHC complexes on its surface [7–11]. It is through interactions with pMHC ligands that T cells become activated and differentiate into effector T cells, which elicit immune responses. Thus, in order to study the conditions under which T cells initiate a response, one needs to understand the dynamics of TCR–pMHC binding.

T cells are derived from precursor cells that migrate from the bone marrow to the thymus, where they rearrange their receptor genes and generate a unique (clonotypic) TCR. In the thymus, immature T cells (or thymocytes) are exposed to an antigenic micro-environment orchestrated by APCs of different types [12] that subject thymocytes to a ‘double test’ by displaying a wide range of pMHC complexes, with peptides derived from household proteins (self-peptides or self-pMHC complexes). Owing to the stochastic nature of the gene rearrangements [13,14], some TCRs will not be able to recognize a self-pMHC ligand (TCRs that are not functional). Other TCRs will recognize it too well, and could give rise to mature T cells with the potential to generate autoimmune responses. Thus, the need for a thymic double test to check the functionality of a thymocyte (positive selection) and its state of tolerance, so that it does not recognize self-pMHC complexes with high affinities (negative selection) [15]. Thymic selection allows only 2–5% of all thymocytes to survive and migrate to the peripheral sites of the immune system (lymph nodes, spleen, etc.) [16,17], where they continuously recirculate via the blood, surveying the antigenic environment displayed once again by APCs.

TCR–pMHC binding events determine T cell responses (survival, proliferation, differentiation or death) at different times and locations during the life of a T cell [18]. For example, Naeher *et al*. [19] have made use of a photo-affinity labelling system (that allows quantitative analysis of pMHC monomer binding to TCR) to show that MHC class I restricted TCRs exhibit an affinity threshold during negative selection. In the light of these results, it is natural to consider the question [20]: how does the number of TCR–pMHC-bound complexes relate to the outcome of negative selection? Current evidence suggests that both the duration and the number of TCR–pMHC bindings play a role [21–23]. Valitutti *et al*. [24,25] have experimentally shown that a few hundred pMHC molecules can serially bind thousands of TCRs. Finally, Sykulev *et al*. [26] and Davis and colleagues [27,28] have provided experimental data suggesting that a few agonist pMHC ligands (5–10) are sufficient to elicit a T cell response. This body of work provides support for the following two hypotheses: (i) TCR–pMHC engagement needs to be sufficiently long to result in productive signal transduction [29] and (ii) T cells can integrate signals; that is, counting devices are at work in T cells to allow signal accumulation, decoding and translation into biological responses [25]. These ideas have been explored by different groups: Palmer and Naeher have provided a biophysical basis for their *affinity threshold for negative selection* hypothesis [19,20], Dushek *et al*. [30] have developed a mathematical ‘productive hit rate model’, Chakraborty and colleagues [31–33] have developed statistical models of how T cells convert analogue inputs into digital outputs and van den Berg & Rand [34] have introduced the idea of a mean triggering rate. The first set of authors introduces the concepts of *dwell time of individual TCR–pMHC complexes* and *productive TCR interactions*, and compute the number of TCR–pMHC interactions required as a function of the TCR–pMHC complex half-life, for a given choice of dwell time and number of productive TCR interactions (see fig. 3 of Palmer & Naeher [20]). Current experimental evidence supports values of dwell time, *τ*, of around 4 s and number of productive TCR interactions, *N*, below 100 [20,35]. In this study, we make use of these ideas to provide a stochastic T cell response criterion based on a mathematical model of TCR–pMHC molecular interactions. The dynamics of a small number of TCR–pMHC binding events, as suggested by the experimental evidence mentioned earlier, is naturally described as a stochastic process, without the need to assume that TCR–pMHC association/dissociation kinetics has reached thermal equilibrium [36–41].

TCR–pMHC binding, and receptor–ligand binding in general, is described by reaction kinetics, assuming that the ligand is in solution and that receptors are membrane-bound on T cells [42,43]. The kinetics is governed, for a given choice of receptor and ligand, by two rates, *k*_+_ and *k*_−_, that give the probability per receptor and per unit of time of a binding and an unbinding event, respectively [36–39,44]. The study of reaction kinetics for the system A + B ⇋ C is not only limited to the case of receptor–ligand interactions, but is of wide interest and has been applied to other problems, such as crystal growth, gene clustering, cellular metabolism and catalytic efficiency of enzyme reactions [45–51]. From a thermodynamic perspective, it is natural to assume that, if one waits long enough, forward (association) and backward (dissociation) reactions reach a steady-state or equilibrium [34]. Thus, in §3, we investigate an equilibrium dynamics model of TCR–pMHC association/dissociation. One candidate T cell response criterion that will be explored is that the timescale of a T cell response correlates with the time a threshold number, *N*, of TCRs must be occupied at equilibrium, *T*_*N*_. However, given the experimental support behind the hypothesis that a few agonist pMHC ligands can suffice to trigger T cell responses [3,26,52] and Palmer's affinity threshold for negative selection [19,20,30], a stochastic approach seems more appropriate [38,39,53]. Furthermore and as discussed earlier, (i) sufficiently long TCR–pMHC engagements are required to initiate the signalling cascade, resulting in productive signal transduction [35], and (ii) T cells can integrate signals; that is, counting devices are at work in T cells to allow signal accumulation, decoding and translation into biological responses [25,23].

With this experimental and theoretical evidence in mind, we explore a different criterion, namely that T cell responses take place once a given number of TCRs (and not necessarily in a simultaneous way), *N*, have been engaged with ligand for at least a dwell time, *τ*, each. The stochastic criterion requires counting the number of productive bindings (a binding is productive if it lasts longer than the threshold dwell time, *τ*, to capture the essence of the signalling cascade [39]). The first time at which this stochastic criterion is satisfied (a first passage time (FPT), as considered from a stochastic process point of view [54]) will be referred here, and in a biological context, to as the (first) time to signal initiation (TSI). We will derive expressions for its mean value, or *mean time to signal initiation* (MTSI), *T*(*N*, *τ*), as a function of *N*, the number of productive TCR–pMHC engagements and *τ*, the dwell time, and its variance. We study the MTSI for different pMHC ligands, thymocytes and temperatures (both association and dissociation rates are temperature-dependent). We make use of recent data [19,55,56] to compare the equilibrium criterion versus the MTSI criterion, to explore the affinity threshold hypothesis and to confront two-dimensional and three-dimensional binding data with the stochastic criterion.

The study has the following structure: §3 describes the main results and in §4 we explore the immunological consequences of the results. Finally, in §5, we provide the mathematical details of the stochastic model developed, and how to derive the deterministic model as a limit of the stochastic model. We also provide analytical expressions for the mean and the variance of the TSI, as well as for the time to reach a threshold number *N* of receptor–ligand complexes, *T*_*N*_.

## 3. Results

### 3.1. Receptor–ligand binding dynamics

Our model of receptor–ligand binding is motivated by the experiments of Palmer and colleagues [19,20], measuring binding of soluble, monomeric pMHC ligands to live thymocytes from T1 TCR transgenic mice. The binding and unbinding reactions can be represented as follows:


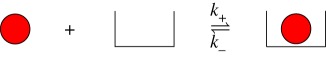


where the *circle* represents a free ligand pMHC and the open *box* an unbound TCR.

We consider two different subsets of TCR transgenic T1 T cells (monoclonal TCR): pre-selection double positive thymocytes (DPs) and mature single positive thymocytes (SPs) [19]. DPs express on average *N*_R_ = 3000 TCRs and SPs express on average *N*_R_ = 30 000 TCRs on their surface. In the experiments, a panel of pMHC ligand complexes is used [19]. Here, we restrict ourselves to three, denoted 4P, 4A and 4N, whose binding parameters are given in table 1. The negatively selecting ligand, 4P, has the highest complex half-life, *t*_1/2_, and lowest equilibrium dissociation constant, *K*_*d*_ [44]. We note that the parameters *K*_*d*_, *t*_1/2_ and *k*_on_ have been introduced and defined in §5.2. The ligand denoted 4N is positively selecting, with the lowest *t*_1/2_ and highest *K*_*d*_. The ligand denoted 4A is called a threshold ligand [19]; it can act as a positively selecting or negatively selecting ligand depending on its concentration [19]. For each ligand type and for a given temperature, the mathematical models require the association and dissociation rates for the TCR–pMHC interaction, *k*_±_ (see §5 and Lauffenburger & Linderman [44]).

**Table 1.** A summary of binding data [19,20]. All constants have been introduced and defined in §5.

cell type	ligand	*K*_*d*_ (M)	*t*_1/2_ (s)	*k*_on_ (s^−1^ M^−1^)	*k*_+_ (s^−1^)	*k*_−_ (s^−1^)
SP thymocyte	4P at 37°C	1.1 × 10^−7^	41	153 691	5.1230 × 10^−10^	0.0169
SP thymocyte	4A at 37°C	5.5 × 10^−6^	0.8	157 533	5.2511 × 10^−10^	0.8664
SP thymocyte	4N at 37°C	5.8 × 10^−5^	0.08	149 385	4.9795 × 10^−10^	8.6643
DP thymocyte	4P at 37°C	8.8 × 10^−8^	39	201 966	6.7322 × 10^−10^	0.01778
DP thymocyte	4A at 37°C	2.2 × 10^−6^	0.79	398 161	1.3272 × 10^−9^	0.8760
DP thymocyte	4N at 37°C	2.9 × 10^−5^	0.23	105 719	3.5240 × 10^−10^	3.0658

Our criterion is that T cell responses are initiated by discrete (stochastic) events and not by attaining the state of thermal equilibrium or steady-state. In order to support our criterion, in §3.2, we explore a stochastic model of receptor–ligand binding, and in §3.3 we analyse the deterministic limit of the mathematical model. The distinction between deterministic and stochastic approaches is not solely a mathematical one, but a choice that has its roots in the biochemical distinction between equilibrium and non-equilibrium dynamics.

### 3.2. Stochastic criterion for T cell responses

We first study the possibility that immunological responses of T cells are determined by the number of TCRs engaged for a minimum threshold time [20]. In order to explore this scenario, we develop a stochastic model of TCR binding to pMHC ligand, described in §5.1. We are interested in calculating the time it takes a T cell to reach (for the first time and not necessarily in a simultaneous fashion) *N* TCR engagements with pMHC ligands, such that each engagement lasts at least a dwell time *τ*. This time defines the stochastic criterion of T cell responses and is denoted in what follows the *first time to signal initiation* (or TSI). The first time to signal initiation (or time to signal initiation, for short) is analogous to a FPT defined in stochastic dynamical systems [49,57]. We note the following implicit assumptions of the stochastic criterion: binding events, as well as unbinding events are considered independent and identically distributed random variables [58,59], and the times to bind, as well as the times to unbind are considered exponentially distributed random processes (see §5.1 for mathematical definitions and choice of notation). In §5.1, we provide analytical expressions for the mean and the variance of the time to signal initiation.

**Figure 1.**
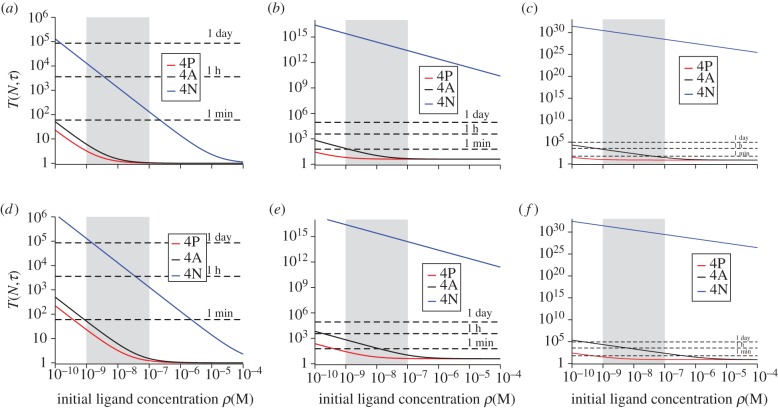
Mean time to signal initiation (MTSI) for a T cell according to the stochastic criterion. MTSI, *T*(*N*,*τ*), for single positive thymocytes (SP), T1 TCRs at 37 degrees. Different panels stand for different pairs of values (*N*,*τ*): (*a*) for (10,1), (*b*) for (10,4), (*c*) for (10,8), (*d*) for (100,1), (*e*) for (100,4) and (*f*) for (100,8). Time units are seconds. The physiologically relevant range of initial ligand concentration is shaded grey, and the dotted lines correspond to a time scale of 1 min, 1 h and 1 day, respectively.

We now make use of the T1 TCR data described in Naeher *et al*. [19] for three different pMHC ligands (4P negatively selecting ligand, 4A threshold ligand, 4N positively selecting ligand) and summarized in table 1. In figure 1, we plot *T*(*N*,*τ*) for SP thymocytes, as a function of the initial ligand concentration, for different values of *N* and *τ* (see §5.1 for mathematical definitions and choice of notation).

From figure 1 (supported by equation (3.1)), we note the following properties of the MTSI.

— For any value of the initial ligand concentration, and for any choice of *N* and *τ*, the stochastic criterion yields the shortest time to respond to the ligand 4P and the largest time to respond to the ligand 4N, in agreement with the experimental data of Naeher *et al*. [19]. Furthermore, 4A (or threshold ligand) displays in all cases (figure 1) an intermediate behaviour. Thus, the MTSI provides qualitative support to the affinity threshold hypothesis introduced in Naeher *et al*. [19]. Given an initial ligand concentration, a choice for (*N*, *τ*) and a time *T* for a T cell response, the positively selecting ligand will not be able to initiate a response within a immunologically relevant time.
— There is no reversion of the hierarchy of ligands as a function of the initial ligand concentration.
— An increase in either *N* or *τ* increases the value of MTSI.
— As the temperature increases, the MTSI also increases (data not shown).

We now proceed to present our results in greater detail.

#### 3.2.1. For single positive thymocytes the mean time to signal initiation is shorter when binding the negatively selecting ligand

We have considered different scenarios, firstly for SP thymocytes [20,35]: (*a*) *N* = 10 bindings and *τ* = 1 s, (*b*) *N* = 10 bindings and *τ* = 4 s, (*c*) *N* = 10 bindings and *τ* = 8 s, (*d*) *N* = 100 bindings and *τ* = 1 s, (*e*) *N* = 100 bindings and *τ* = 4 s and (*f*) *N* = 100 bindings and *τ* = 8 s. We compare the mean T cell response times (MTSI) of a positively selecting ligand (4N), a threshold ligand (4A) and a negative selecting ligand (4P), for varying initial ligand concentrations for the TCR system T1. As seen in figure 1, the negative-selecting ligand is characterized by the shortest MTSI in all cases considered. This is a result of both its higher *k*_on_ and lower *k*_off_, which means bindings occur more frequently and are more likely to last longer. This also accounts for the more pronounced differences in MTSI when either the threshold time, *τ*, is increased from 1 to 8 s or when the number of bindings required, *N*, is increased from 10 to 100.

A consequence of the stochastic criterion is that, the larger the number of bindings required, *N*, the less important stochastic effects become. In this case, the coefficient of variation tends to zero as *N* increases (see equation (5.17)). Immunologically, this is relevant as the criterion relegates *N* to a role secondary to that of the dwell time *τ*. (For example, compare panels *a* and *b* (change in *τ*) and panels *a* and *d* (change in *N*) in figure 1.)

### 3.3. Equilibrium time to *N* TCR–pMHC complexes

**Figure 2.**
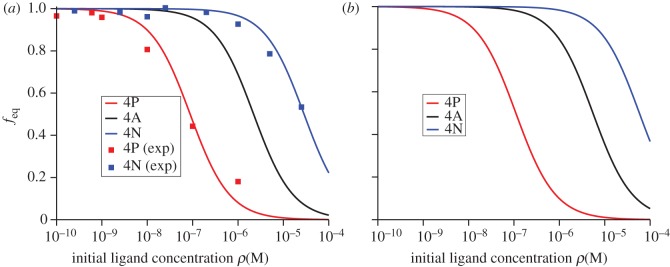
Fraction of unbound T cell receptor at equilibrium, *f*_eq_, computed making use of the equations derived in §5.2. (*a*) Double positive thymocytes. (*b*) Single positive thymocytes. Squares are the experimental measured values provided in Naeher *et al*. [19].

If one was to assume that ligand hierarchy or potency (for a given TCR) is determined by how rapidly thermal equilibrium of TCR–pMHC complexes is established, it would be natural to compare the dose–response binding curves for the different ligands under consideration [19]. In figure 2, we plot *f*_eq_ (see §5.2), the fraction of free TCR molecules at equilibrium, as a function of the initial ligand concentration (dose–response) for different ligands and for DPs (*a*) and SPs (*b*). These curves, which have been computed making use of the equations derived in §5.2, agree with the experimental equilibrium binding curves of Naeher *et al*. [19].

**Figure 3.**
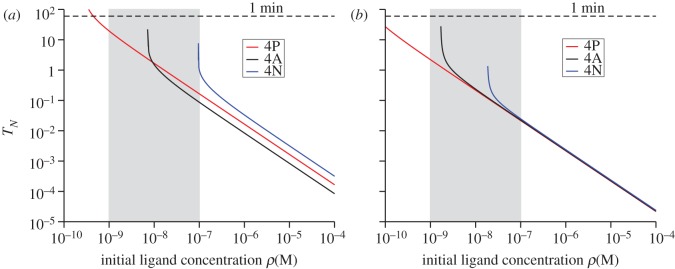
Equilibrium time, *T*_*N*_, with *N* = 10, using the deterministic model of §5.2. (*a*) Double positive thymocytes. (*b*) Single positive thymocytes. Time units are seconds. The physiologically relevant range of initial ligand concentration is shaded grey.

We now continue to explore the equilibrium dynamics of receptor–ligand binding with a second T cell response criterion. We introduce *T*_*N*_, the time needed to reach *N* TCR–pMHC complexes. In §5.2, we derive an expression for *T*_*N*_ from the solution of an ordinary differential equation [44]. This criterion is, to some extent, the deterministic version of the stochastic criterion that we have introduced in the previous section. In figure 3, we show the time to reach *N* = 10 TCR–pMHC complexes for both double and single positive thymocytes (figure 3*a* and *b*, respectively).

Inspection of both the DP and SP thymocytes cases (figure 3*a* and *b*, respectively), clearly indicates that this second equilibrium criterion does not allow discrimination between ligands for intermediate to high initial ligand concentrations. That is, for a large range of initial concentrations, this criterion cannot distinguish between positively and negatively selecting ligands, contradicting what has been experimentally found in Naeher *et al*. [19]. Thus, this criterion cannot account for the hierarchy of activity of peptides observed *in vivo* [19]. Furthermore, for low enough concentrations, there is an abrupt change in the behaviour of the ligands 4N and 4A, as *T*_*N*_ becomes unbound, that is, for these two ligands, *T*_*N*_ becomes uncontrollably large, and for small initial concentrations these two ligands will not reach *N* TCR–pMHC complexes in a finite amount of time. This behaviour shifts towards higher concentrations as the value of *N* increases. The immunological implications of this criterion are: for high concentrations, all the ligands elicit the same response and thus *T*_*N*_ behaves as an *all-or-nothing* T cell response mechanism. If we were to include the condition that each TCR–pMHC complex needs to remain bound for a minimum dwell time (defined later as *τ*), this would only shift the curves by an amount *τ* vertically.

In the case of DP thymocytes, figure 3*a*, the implications of this equilibrium criterion are even more disappointing as the roles of 4P and 4A are reversed and the threshold ligand 4A becomes the negatively selecting ligand. Both implications are in disagreement with the hierarchy of ligands experimentally determined [19,20].

From an immunological perspective, and as a function of the initial concentration of ligand, one expects the following behaviour [19,20]: (i) for large enough concentrations (above the physiological range), negatively selecting ligands remain negatively selecting, positively selecting ligands remain positively selecting and threshold ligands become negatively selecting, and (ii) for physiological concentrations, negatively selecting ligands remain negatively selecting, positively selecting ligands remain positively selecting and threshold ligands remain threshold. We can conclude that this behaviour is well characterized by the stochastic criterion introduced in §3.2 (see, figure 1*b*,*c* or figure 6*b*,*c*) but is at odds with the equilibrium criterion discussed in this section.

### 3.4. Statistics of the mean time to signal initiation

**Figure 4.**
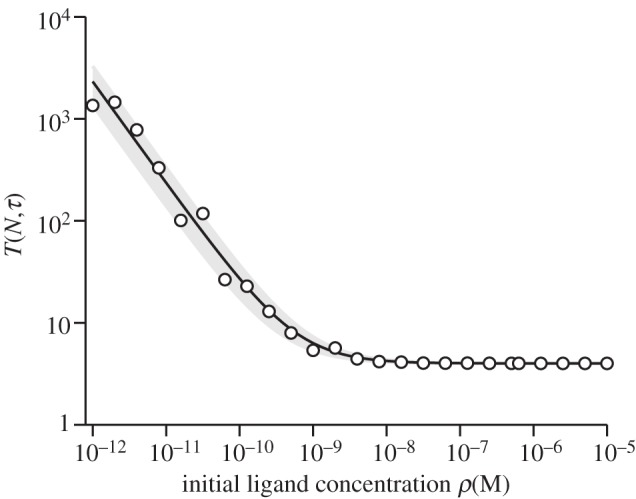
Mean time to signal initiation, *T*(*N*,*τ*), for *N* = 10 and *τ* = 4 s, as a function of the initial ligand concentration. The ligand is 4P and the T cells are SPs. The solid line represents *T*(*N*,*τ*), calculated with the analytical expression (3.1), and the shaded area is one standard deviation, calculated with the analytical expression (3.2). The open circles represent *T*(*N*,*τ*), numerically calculated with just one computational run (see §5.4). Time units are seconds.

Here, we introduce the equation for the time it takes a T cell to reach (for the first time and not necessarily in a simultaneous fashion) *N* TCR engagements with pMHC ligands, such that each engagement lasts at least a dwell time *τ*. We also explore the implications of this equation for the MTSI (which is in the theory of stochastic processes a mean FPT [58]), *T*(*N*,*τ*), as a function of its relevant parameters (at a given temperature). The relevant parameters are the initial pMHC ligand concentration, *ρ*, the average number of TCRs expressed on the surface of a T cell, *N*_R_, and the association and dissociation rates for a given pMHC ligand. As derived in §5.3, the MTSI is given by3.1
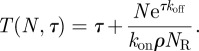
The previous equation can be intuitively understood as follows. The probability that, once engaged, a receptor stays engaged for at least a time *τ* is exp(−*τk*_off_). The mean number of complexes formed such that *N* of them remain bound for longer than *τ* is *N*′ = exp(*τk*_off_)*N*. Note that *T*(*N*,*τ*) is always a decreasing function of *ρ* and *k*_on_, but an increasing function of *k*_off_. In the limit of very small initial ligand concentration, we have 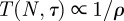. In the limit of large initial ligand concentration, we have 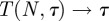. In §5, we have also derived the following analytical expression for the variance of the MTSI, as a function of *N* and *τ*:3.2
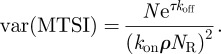
The predictions of (3.1) are in excellent agreement with the exact numerical simulations (see §5) as can be seen in figure 4, which provides a comparison between the numerical simulations and the theoretical predictions of (3.1) and (3.2).

**Figure 5.**
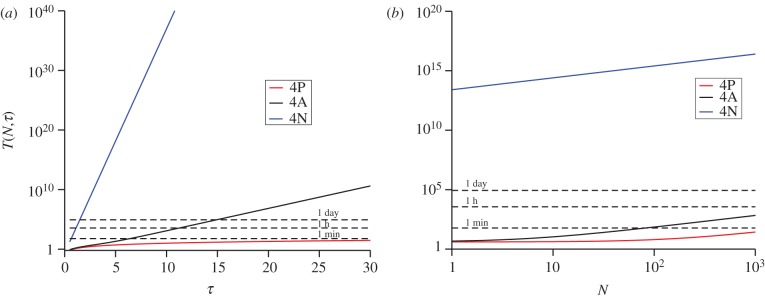
Mean time to signal initiation according to the stochastic criterion. MTSI, *T*(*N*,*τ*), for single positive thymocytes (SP), T1 TCRs at 37 degrees. (*a*) MTSI as a function of *τ* with *N* = 10 and (*b*) MTSI as a function of *N* with *τ* = 4 s. (*a*,*b*) The initial ligand concentration has been set equal to *ρ* = 10^−8^ M. Time units are seconds.

In figure 5, we explore the dependence of *T*(*N*,*τ*), given an initial ligand concentration, as a function of *τ* (for fixed *N*) and as a function of *N* (for fixed *τ*). The left panel (figure 5*a*) illustrates the dramatic difference in *T*(*N*,*τ*) based on small differences in *k*_off_. As predicted by equation (3.1), *T*(*N*,*τ*) depends linearly on *N* (figure 5*b*).

We conclude this section with a final application of the stochastic criterion to the problem of ligand self–non-self discrimination. In the spirit of the example discussed by van der Merwe & Dushek [35] (box 1), we study the implications of the MTSI formula to illustrate the possibility of self–non-self discrimination. Suppose that ligand F ‘foreign’ has *k*_off_ = 1.0 s^−1^ and ligand S ‘self’ has *k*_off_ = 5.0 s^−1^, that both have the same value of *k*_on_, but the concentration of S is 100 times that of F. If *k*_on_*N*_R_*ρ* = 10 s^−1^ for F, and if *k*_on_*N*_R_*ρ* = 1000 s^−1^ for S, then *T*(10, 4) = 59 s for F and *T*(10, 4) = 5 × 10^6^ s for S. In this example, the stochastic criterion produces very clear self–non-self discrimination.

### 3.5. Pre-selection DPs versus SPs

**Figure 6.**
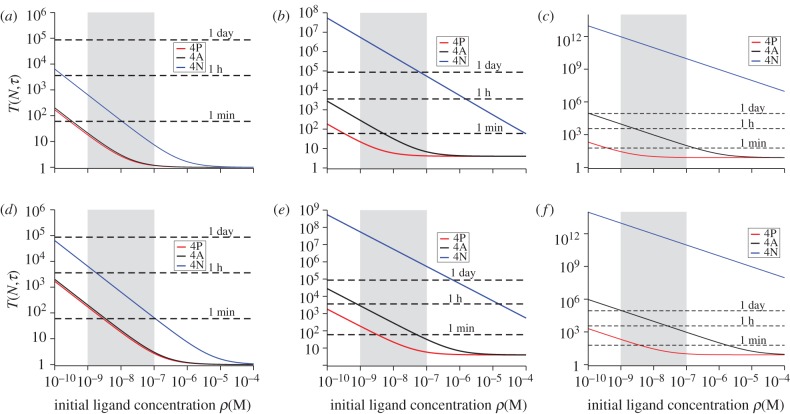
Mean time to signal initiation (MTSI) for a T cell according to the stochastic criterion. MTSI, *T*(*N*, *τ*), for double positive thymocytes (DP), T1 TCRs at 37 degrees. Different panels stand for different pairs of values (*N*, *τ*): (*a*) for (10, 1), (*b*) for (10, 4), (*c*) for (10, 8), (*d*) for (100, 1), (*e*) for (100, 4) and (*f*) for (100, 8). Time units are seconds. The physiologically relevant range of initial ligand concentration is shaded grey, and the dotted lines correspond to a time scale of 1 min, 1 h and 1 day, respectively.

We now explore the MTSI hypothesis on pre-selection DP thymocytes, which have 10-fold lower average number of TCRs on their surface than SP thymocytes. We also note that the binding rates (for a fixed temperature) are different for SP and DP thymocytes (table 1). We have made use of the three different ligands (4P, 4A and 4N) of the T1 TCR system [19]. Our results are summarized in figure 6. DPs are not as capable of discriminating between negatively selecting and threshold ligands as SPs at low initial pMHC ligand concentrations.

As can be seen from plots (*a*) and (*d*), with *τ* = 1 s, the stochastic criterion does not distinguish between 4P (negatively selecting) and 4A (threshold). The ability to discriminate at 37 degrees improves if we choose *τ* = 4 s, (figure 6*b*,*e*). These results suggest that if negative selection happens too early (at the pre-selection DP stage), it might not be effective, as pre-selection DPs will not distinguish between signals delivered by positively selecting ligands and negatively selecting ligands. Threshold ligands, such as 4A, are expected to behave as positively selecting ligands at low concentration, but as negatively selecting ligands at high concentration, as experimentally shown [19,20].

### 3.6. Two-dimensional binding data: stochastic T cell response criterion

Single-molecule microscopy and fluorescence resonance energy transfer [55], as well as adhesion frequency and thermal fluctuations assays [56] are recent alternative methods of quantifying TCR–pMHC binding, with the advantage that both molecules are anchored on cell surfaces [35,55,56,60]. Recent experimental kinetic parameters (such as *k*_on_ and *k*_off_), measured in such two-dimensional conditions [55,56], are given in tables 2 and 6.

**Table 2.** A summary of *in situ* (two-dimensional) binding data taken from Huppa *et al*. [55] from the experimental CD4^+^ 2B4 TCR mouse model.

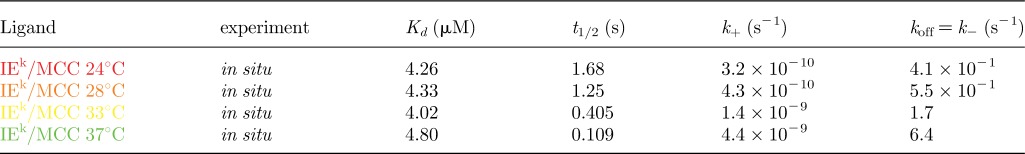

In this section, we explore the stochastic criterion in light of these recent two-dimensional measurements. Note that the stochastic criterion allows us to consider both scenarios (two- and three-dimensional), as follows.

- For two-dimensional binding (receptor and ligand molecules anchored on cell membranes), with number densities of receptor and ligand *M*_R_, *M*_L_, respectively, *A*_c_ the area of contact between the cells, and *k*_on_^(two-dimensional)^ and *k*_off_^(two-dimensional)^ the two-dimensional on and off rates, respectively, the MTSI is given by3.3
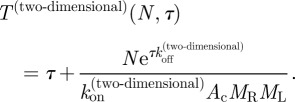
- For three-dimensional binding (receptor molecules anchored on cell membrane and ligand in solution), with ligand concentration *ρ*, number of receptors on the cell surface *N*_R_ and *k*_on_^(three-dimensional)^ and *k*_off_^(three-dimensional)^ the three-dimensional on and off rates, respectively, the mean MTSI is given by3.4
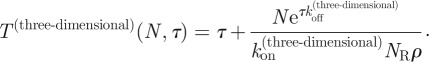

We make use of the stochastic criterion with experimental data, measured in a two-dimensional context, from earlier studies [55,56]. Let us assume that given a TCR, ligand potency correlates inversely with the value of *T*(*N*,*τ*), that is, the most potent ligand for the given TCR is that with the smallest value of the MTSI. We note that for a chosen value of *τ*, the ligand hierarchy does not depend on *N*, as can be seen from (3.3) and (3.4).

The experimental set-up of Huppa *et al*. [55] is for the CD4^+^ 2B4 TCR mouse model and for the IE^k^/MCC ligand at different temperatures. We have considered their binding data for different temperatures and both *in situ* and *in vitro* conditions [55] (tables 2 and 3). The stochastic criterion (with *N* = 10 and *τ* ∈ [1,10] s) has been used to rank the experimental data (fixed ligand at different temperatures) according to their MTSI values (tables 4 and 5). If we assume that the MTSI correlates with the time of a T cell response, and thus, with the potency of the ligand,^1^ the stochastic criterion implies that it is only for *τ* > 5 s when both *in situ* (two-dimensional) and *in vitro* (three-dimensional) binding data agree on ligand potency (or hierarchy). In this case, and for *τ* > 5 s, it is the lowest temperature that yields the shortest MTSI, and as the temperature is increased, the MTSI increases. A plausible way to reconcile both *in situ* and *in vitro* data is to hypothesize that the early intracellular molecular steps of the T cell signalling cascade (a kinetic proof-reading mechanism [52,61–63]), require at least a time *τ* > 5 s.

**Table 3.** A summary of *in vitro* (three-dimensional) binding data taken from Huppa *et al*. [55] from the experimental CD4^+^ 2B4 TCR mouse model.

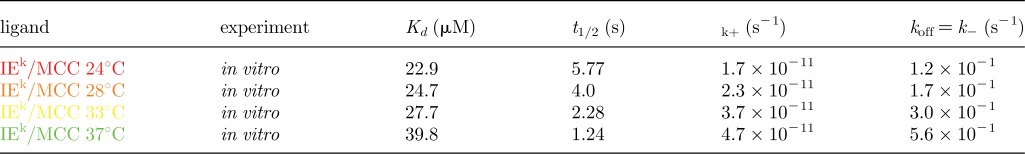

**Table 4.** Each row is the ligand ranking, for a given value of *τ*, according to the stochastic criterion for the parameters in table 2. The first ranking ligand corresponds to the shortest MTSI. The parameters and colour scheme correspond to the *in situ* experiments of table 2.

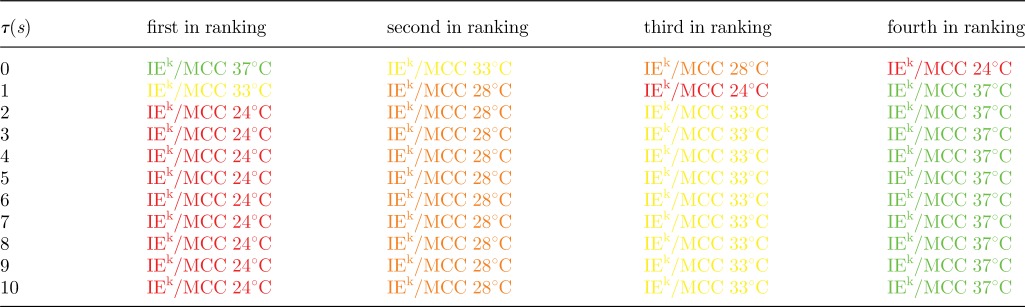

**Table 5.** Each row is the ligand ranking, for a given value of *τ*, according to the stochastic criterion for the parameters in table 2. The first ranking ligand corresponds to the shortest MTSI. The parameters and colour scheme correspond to the *in vitro* experiments of table 3.

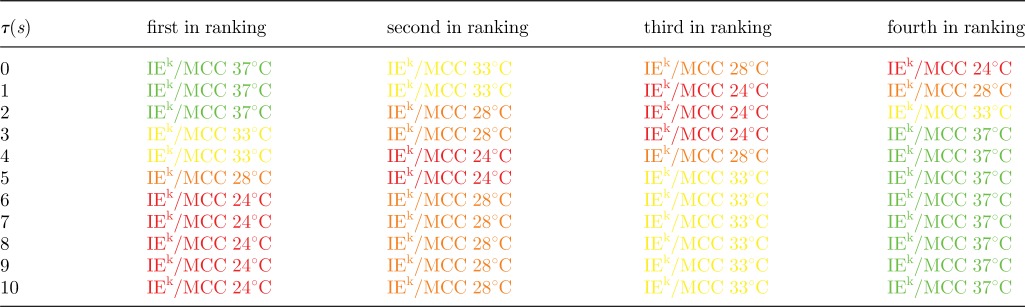

The experimental set-up of Huang *et al*. [56] is for the CD8^+^ OT1 TCR mouse model and six different ligands. We have considered their two-dimensional binding data [56] (table 6), and the three-dimensional binding data of Gascoigne *et al*. [64] (table 7). The stochastic criterion (with *N* = 10 and *τ* ∈ [1,10] s) has been used to rank the experimental data (six different ligands at a given temperature) according to their MTSI values (tables 8 and 9). If we assume that the MTSI correlates with the time of a T cell response, and, thus, with the potency of the ligand, the stochastic criterion suggests that the two-dimensional and three-dimensional hierarchies are very different. The three-dimensional hierarchy does not change, except for a switch at 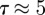 s, when V-OVA and R4 interchange their rankings. On the other hand, the two-dimensional hierarchy depends a lot on the value of *τ*. We note that the hierarchy of ligands for *τ* < 1 s is almost an inversion of the hierarchy that gets established for values of *τ* greater than 6 s. This is not surprising, given the negative correlation between two-dimensional and three-dimensional off-rates reported by Huang *et al*. [56].

**Table 6.** A summary of two-dimensional binding data taken from Huang *et al*. [56] for the CD8^+^ OT1 TCR experimental mouse model.

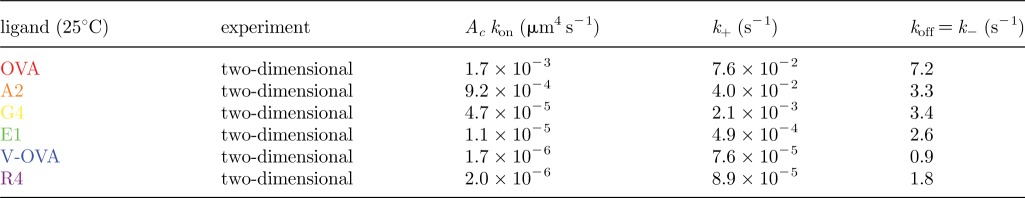

**Table 7.** A summary of three-dimensional binding data taken from [64] for the CD8^+^ OT1 TCR experimental mouse model.

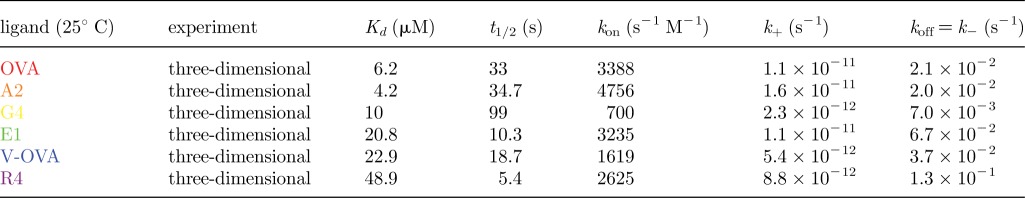

**Table 8.** Each row is the ligand ranking, for a given value of *τ*, according to the stochastic criterion for the parameters in table 6. The first ranking ligand corresponds to the shortest MTSI. The parameters and colour scheme correspond to the two-dimensional experiments of table 6.

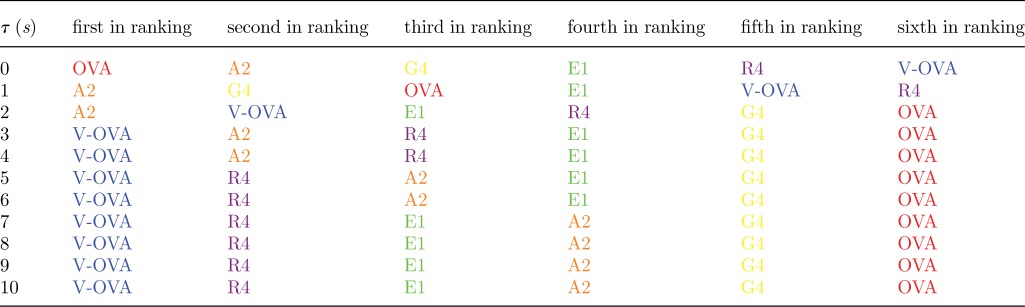

**Table 9.** Each row is the ligand ranking, for a given value of *τ*, according to the stochastic criterion for the parameters in table 7. The first ranking ligand corresponds to the shortest MTSI. The parameters and colour scheme correspond to the three-dimensional experiments of table 7.

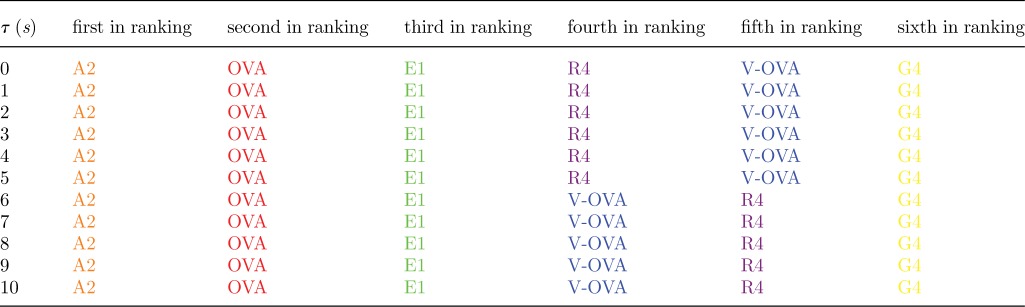

## 4. Discussion

T cell receptor binding to ligand determines T cell responses at different times and locations during the life of a T cell [15]. Current experimental evidence, as reviewed in Valitutti *et al*. [25], provides support to the following: (i) sufficiently long receptor–ligand engagements are required to initiate the T cell signalling cascade that results in productive signal transduction, and (ii) counting devices are at work in T cells to allow signal accumulation, decoding and translation into biological responses. In other words, both the duration and number of TCR–pMHC bindings play a role in T cell responses [21,30]. These ideas have already been explored by Palmer & Naeher [20] in order to provide a biophysical basis for their *affinity threshold for negative selection* hypothesis [19]. The authors introduce the concepts of *dwell time of individual TCR–pMHC complexes* and *productive TCR interactions*, and compute the number of TCR–pMHC interactions required as a function of the TCR–pMHC complex half-life, for a given choice of dwell time and number of productive TCR interactions (see fig. 3 of Palmer & Naeher [20]). In light of these results, and the fact that in the thymus SP thymocytes only have 4–5 days to scan the medullary environment [12,65,66] and in the periphery the dose- and time-dependence of antigen localization determine whether protective immunity is induced or not [67], in this study we have explored, with mathematical models, the timescales associated with T cell responses. We have considered two different criteria: a stochastic one—(i) the mean time (or mean FPT) it takes to have had *N* receptor–ligand complexes bound for at least a dwell time, *τ*, each— and one based on equilibrium—(ii) the time a threshold number, *N*, of TCRs are occupied at equilibrium. The dynamics of a small number of TCR–pMHC binding events is naturally described as a stochastic process, without the need to assume that TCR–pMHC association/dissociation kinetics has reached thermal equilibrium [36–40]. We have applied both the deterministic and stochastic criteria to the experimental data presented in Naeher *et al*. [19]. Our results indicate that the stochastic criterion provides support to the thymic affinity threshold hypothesis suggested by Palmer [19,20], whereas the equilibrium one does not. Thus, we propose as a timescale associated to T cell responses: the first time at which the stochastic criterion is satisfied, which is referred to as an FPT in the theory of stochastic processes [58], but has been referred to as the first time to signal initiation in this study. Furthermore, other properties of the stochastic criterion are (i) for the values of *N* and *τ* considered, the calculated MTSIs are of the order of the timescales associated to negative selection and T cell activation, (ii) a very small number (fewer than 10) of cognate ligand molecules is sufficient to elicit a T cell response, which is consistent with the serial engagement model, (iii) it provides an intuitive way to understand self–non-self discrimination, (iv) it relegates *N* to a secondary role to that of the dwell time, *τ*, which is consistent with the kinetic proof-reading model, and (v) it can be applied to either two- or three-dimensional binding data. Our results indicate that for the experimental data of Huppa *et al*. [55] one can identify a threshold dwell time, *τ*, for TCR–pMHC complexes of 5 s that can account for the same ligand hierarchy for both *in situ* and *in vitro* data. For the data reported by Huang *et al*. [56], we note that the two-dimensional hierarchy, as determined by the stochastic criterion, is extremely sensitive to the value of *τ*: the hierarchy of ligands for *τ* < 1 s is almost an inversion of the hierarchy that gets established for values of *τ* greater than 6 s. This is not surprising, given the negative correlation between two-dimensional and three-dimensional off-rates reported by the authors.

The previous discussion also stresses one of the main results of this study, namely that stochastic effects are important in the timescales that determine cellular responses: during thymic negative selection [12,65,66] or during the initiation of T cell responses in the periphery [67].

As discussed in Altan-Bonnet & Germain [52], rapid, sensitive and highly discriminatory TCR-induced signals, yet exquisitely ligand specific, can be explained in terms of a negative feedback (phosphatase mediated), which suppresses signalling by weak ligands, and a positive feedback (ERK mediated), which is induced by strong TCR–pMHC ligands. Furthermore, recent work by Dushek *et al*. [63] has considered the role of TCR–pMHC rebinding, within a kinetic proofreading model [61,62], as a potential mechanism for pMHC ligand discrimination. In this study, we have not attempted to provide a mechanistic derivation of the dwell time, *τ*, introduced in the stochastic criterion. The mechanisms discussed in earlier studies [52,63,68] will allow us to explore different kinetic proofreading scenarios and feedback loops, to justify the origin of *τ*. This and TCR/pMHC diffusion on cellular membranes [69] will be considered elsewhere.

We conclude by summarizing the mathematical results of this study: we have made use of a stochastic model for the binding and unbinding kinetics of receptor–ligand interactions [38], that is a birth and death stochastic process for the number of receptor–ligand complexes. This model has allowed us to formulate the stochastic criterion and to derive analytical expressions for the mean value of TSI, *T*(*N*,*τ*) as a function of *N* and *τ*, and its variance.

## 5. Methods

### 5.1. Stochastic model

We study the dynamics of monovalent receptor binding to monomeric ligand with a stochastic model that is represented as follows:


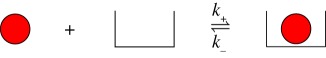


An unbound receptor can bind a free ligand with rate *k*_+_, and an engaged receptor can become dissociated from the ligand with rate *k*_−_. Both *k*_±_ have units of inverse time. At the initial time, *t* = 0, all *M*_R_ receptors are unbound, and there are *M*_L_ free ligands. The stochastic variable **X**_*t*_ represents the number of engaged receptors at time t. Its state space *S* is given by the set 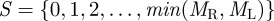. The dynamics of the stochastic variable **X**_*t*_ (number of engaged receptors at time *t*) can be derived from the transition probabilities that prescribe the events that can take place in a small time interval. There are only two types of events:

- an association event that increases the number of engaged receptors by one unit, and
- a dissociation event that reduces the number of engaged receptors by one unit.

The stochastic model for the binding and unbinding of receptors and ligands is a continuous time Markov chain, a birth and death process [58,70] with rates
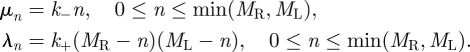


Let *p*_*n*_(*t*) be the probability that there are *n* engaged receptors at time *t*. That is, 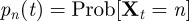. These probabilities satisfy the following system of differential equations [70]:5.1
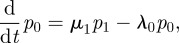

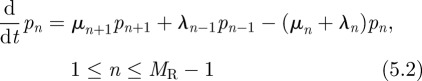
5.3
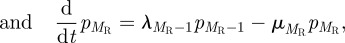
where we have assumed that *M*_R_ ≤ *M*_L_. Similar equations can be derived in the case *M*_R_ > *M*_L_. The mean number of engaged receptors at time *t* is 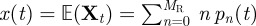.

It obeys the following differential equation:5.4
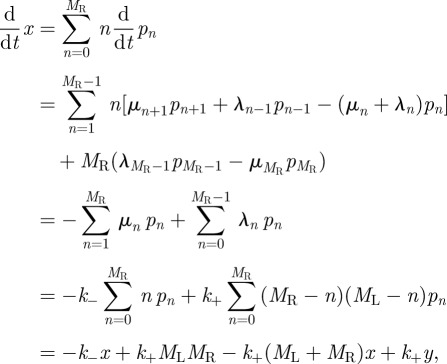


where 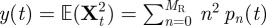.

If the experimental system under consideration has a number, *N*_c_, of T cells, each of them with an average number, *N*_R_, of TCRs on their surface, the mean number of engaged receptors *per T cell* at time *t* is given by5.5
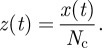
Let us introduce the stochastic variable, **Z**_*t*_, such that **X**_*t*_ = *N*_c_**Z**_*t*_, and the variable 
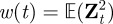
. If we make use of these definitions and the fact that *M*_R_ = *N*_c_*N*_R_, it is easy to derive the following equation for the time evolution of *z*(*t*):5.6
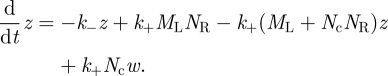


### 5.2. Deterministic approximation

The deterministic approximation consists of neglecting fluctuations in equation (5.6) by setting *w*(*t*) = *z*^2^(*t*), so that d*z*/d*t* = *f*(*z*), where5.7
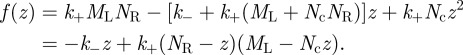
The two solutions of *f*(*z*) = 0 are *z*_1_ and *z*_2_, where5.8
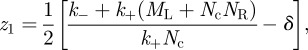
5.9
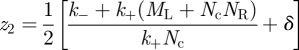
and5.10
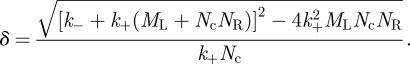


As 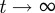, 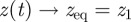, the stable steady-state value [44]. We also introduce the *per T cell* fraction of unbound receptors in the steady-state, given by 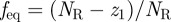. The exact solution of equation (5.6), *z*(*t*), with initial conditions, *z*(0) = 0, is given by
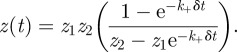
An important quantity that is obtained from this solution is the time to reach *N* TCR–pMHC complexes, that is, *T*_*N*_, such that *z*(*T*_*N*_) = *N*. *T*_*N*_ can be computed to yield:
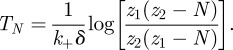


#### 5.2.1. Ordinary differential equation under the assumption of soluble ligand binding

We may rewrite equation (5.6) as
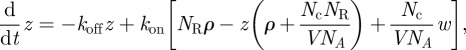
where we have introduced the following parameters:
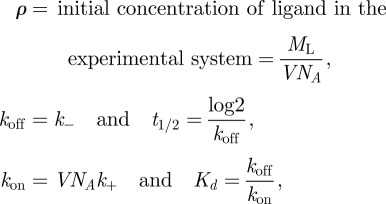
with *V* the volume of the experiment and *N*_*A*_ Avogadro's number.

The deterministic approximation consists of neglecting fluctuations by setting *y*(*t*) = *x*^2^(*t*), or *w*(*t*) = *z*^2^(*t*), so that5.11
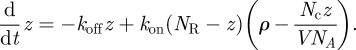
If we introduce
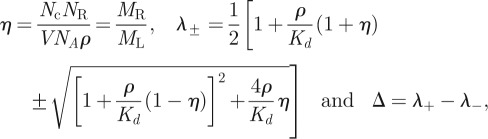
then the solution of equation (5.6), with *z*(0) = 0, is given by5.12
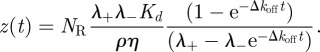
From this solution, one can obtain the equilibrium value of the average number of engaged receptors *per T cell*, *z*_eq_, and *T*_*N*_. These are given by the following expressions:5.13
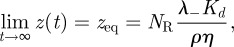
and5.14
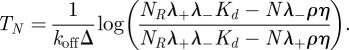


### 5.3. The mean and variance of the first passage time (or first time to signal initiation)

We now suppose that T cell responses take place once *N* TCRs have been engaged with ligand, for at least a time *τ* each. The first time at which this criterion is satisfied is referred to as a FPT [58]. We will derive expressions for its mean value, *T*(*N*,*τ*), and variance.

At the instant of its formation, any ligand–receptor complex has probability 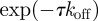 of remaining bound for longer than the dwell time *τ*. A binding that does so is said to be productive. Let 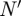 be the mean total number of binding events before the *N*th productive one. We can then write (as binding events are independent)
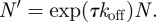
Let *t*_*i*_ be the time that the *i*th ligand–receptor complex is formed. By definition, we have that FPT(*N*, *τ*) = *τ*+ *t*_*N*′_ and thus, 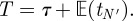 Each of the *N*′ times between binding events, *t*_*i*+1_ − *t*_*i*_, is exponentially distributed and therefore has standard deviation equal to its mean [58]. The time *t*_*N*′_, which is a sum of exponentially distributed random times, therefore has variance proportional to *N*′. If, up to time *t*_*N*′_, the number of bound receptors is much less than *N*_R_ and much less than *ρVN*_*A*_/*N*_*c*_, then *t*_*N*′_ is the sum of *N*′ independent, exponentially distributed random variables with mean (*k*_on_*ρN*_R_)^−1^, that is5.15
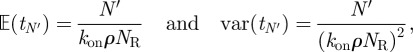
so that5.16
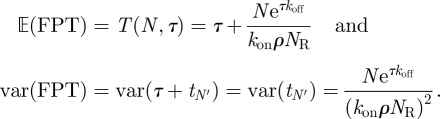
Note that the coefficient of variation of the FPT can be computed as follows:5.17
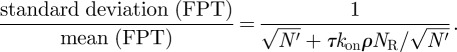


With more generality, we may assume that the initial concentration of ligands, *ρ*, is constant (neglect ligand depletion due to binding), but take into account that the number of occupied TCRs on a T cell is a finite fraction of *N*_R_. The total number of receptors that bind at least once before time *t* is *N*_R_ [1 − exp(−*k*_on_*ρt*)]. Let *B*(*t*) be the mean number of bound receptors at time *t*. Then, we can write5.18
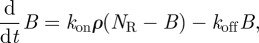
and if we solve for *B*(*t*), we have5.19
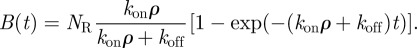
The mean number of binding events up to time *t* is *C*(*t*), where5.20
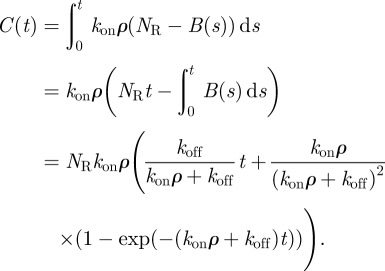
If 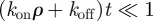, then 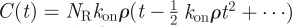. Setting *C*(*T* − *τ*) = *N*^′^ gives the following expression for *T*:
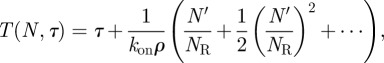
which includes correction terms to the approximation of *T*(*N*, *τ*) calculated in (5.16). Note that the correction terms become negligible when 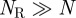. For the values of *N* considered in this study, the correction terms can be neglected, as has been verified in numerical computations.

We conclude by providing, without derivation, the analytical expressions for the mean and the variance of the FPT in a two-dimensional setting. It is easy to show that5.21
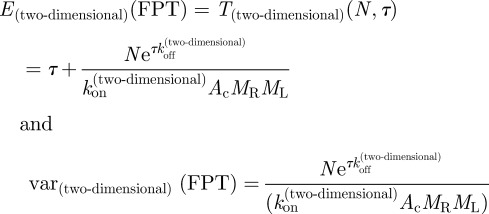


### 5.4. Numerical simulation method

We use the stochastic simulation, or Gillespie algorithm [71,72], where the number of bound ligands as a function of time, in each realization, is explicitly generated. If there are *n* bound ligands at time *t*, then the first event after time *t* is either binding, with probability *λ*_*n*_ /(*λ*_*n*_ + *μ*_*n*_), or unbinding, with probability *μ*_*n*_ /(*λ*_*n*_ + *μ*_*n*_ ). The time at which the event occurs is *t* = *Δt*, where *Δt* is a random variable and 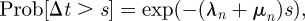 *s* > 0. We study the dynamics of the receptor–ligand system using parameters *k*_+_, *k*_−_, *N*_R_ and *M*_L_ corresponding to the ligands 4P, 4A and 4N. Binding and unbinding times are recorded, and a realization ends when the stochastic criterion (*N* bindings that last, for at least, a time *τ* each) is first satisfied. By averaging over realizations, we are able to compute mean FPTs and coefficients of variation.

## Footnotes

1 We note that in this case, the ligand is kept fixed but the experimental temperature increases (tables 2 and 3).

## References

[1] Coombs D. Kalergis A. Nathenson S. Wofsy C. Goldstein B. 2002 Activated TCRs remain marked for internalization after dissociation from pMHC. Nat. Immunol. 3, 926–931. 10.1038/ni838 (10.1038/ni838) 12244312

[2] Stone J. Chervin A. Kranz D. 2009 T-cell receptor binding affinities and kinetics: impact on T-cell activity and specificity. Immunology 126, 165–176. 10.1111/j.1365-2567.2008.03015.x (10.1111/j.1365-2567.2008.03015.x) 19125887 PMC2632691

[3] Ebert P. Li Q. Huppa J. Davis M. 2010 Functional development of the T Cell receptor for antigen. Prog. Mol. Biol. Trans. Sci. 92, 65–100. 10.1016/S1877-1173(10)92004-8 (10.1016/S1877-1173(10)92004-8) PMC4887107 20800817

[4] Košmrlj A. Jha A. Huseby E. Kardar M. Chakraborty A. 2008 How the thymus designs antigen-specific and self-tolerant T cell receptor sequences. Proc. Natl Acad. Sci. USA 105, 16 671–16 676. 10.1073/pnas.0808081105 (10.1073/pnas.0808081105) PMC2575478 18946038

[5] Lyons D. S. Lieberman S. A. Hampl J. Boniface J. J Chien Y. Berg L. J. Davis M. M. 1996 A TCR binds to antagonist ligands with lower affinities and faster dissociation rates than to agonists. Immunity 5, 53–61. 10.1016/S1074-7613(00)80309-X (10.1016/S1074-7613(00)80309-X) 8758894

[6] Lanzavecchia A. Lezzi G. Viola A. 1999 From TCR engagement to T cell activation: a kinetic view of T cell behavior. Cell 96, 1–4. 10.1016/S0092-8674(00)80952-6 (10.1016/S0092-8674(00)80952-6) 9989490

[7] Yewdell J. Reits E. Neefjes J. 2003 Making sense of mass destruction: quantitating MHC class I antigen presentation. Nat. Rev. Immunol. 3, 952–961. 10.1038/nri1250 (10.1038/nri1250) 14647477

[8] Stevanovic S. Schild H. 1999 Quantitative aspects of T cell activation–peptide generation and editing by MHC class I molecules. Semin. Immunol. 11, 375–384. 10.1006/smim.1999.0195 (10.1006/smim.1999.0195) 10625591

[9] Rammensee H. Falk K. Rötzschk O. 1993 Peptides naturally presented by MHC class I molecules. Annu. Rev. Immunol. 11, 213–244. 10.1146/annurev.iy.11.040193.001241 (10.1146/annurev.iy.11.040193.001241) 8476560

[10] Hunt D. Henderson R. Shabanowitz J. Sakaguchi K. Michel H. Sevilir N. Cox A. Appella E. Engelhard V 1992 Characterization of peptides bound to the class I MHC molecule HLA-A2. 1 by mass spectrometry. Science 255, 1261–1263. 10.1126/science.1546328 (10.1126/science.1546328) 1546328

[11] Tsomides T. Walker B. Eisen H. 1991 An optimal viral peptide recognized by CD8+ T cells binds very tightly to the restricting class I major histocompatibility complex protein on intact cells but not to the purified class I protein. Proc. Natl Acad. Sci USA 88, 11 276–11 280.(10.1073/pnas.88.24.11276) 1722325 PMC53117

[12] Klein L. 2009 Dead man walking: how thymocytes scan the medulla. Nat. Immunol. 10, 809–811. 10.1038/ni0809-809 (10.1038/ni0809-809) 19621041

[13] Janas M. Varano G. Gudmundsson K. Noda M. Nagasawa T. Turner M. 2010 Thymic development beyond β-selection requires phosphatidylinositol 3-kinase activation by CXCR4. J. Exp. Med. 207, 247–261. 10.1084/jem.20091430 (10.1084/jem.20091430) 20038597 PMC2812547

[14] Goldrath A. Bevan M. 1999 Selecting and maintaining a diverse T-cell repertoire. Nature 402, 6–13. 10580495 10.1038/46218

[15] Palmer E. 2003 Negative selection: clearing out the bad apples from the T-cell repertoire. Nat. Rev. Immunol. 3, 383–391. 10.1038/nri1085 (10.1038/nri1085) 12766760

[16] Palmer E. 2006 The T-cell antigen receptor: a logical response to an unknown ligand. J. Recept. Signal Transduct. Res. 26, 367–378. 10.1080/10799890600919094 (10.1080/10799890600919094) 17118787

[17] van Meerwijk J. Marguerat S. Lees R. Germain R. Fowlkes B. MacDonald H. R. 1997 Quantitative impact of thymic clonal deletion on the T cell repertoire. J. Exp. Med. 185, 377–384. 10.1084/jem.185.3.377 (10.1084/jem.185.3.377) 9053438 PMC2196036

[18] Petrie H. Zúñiga-Pflücker J. 2007 Zoned out: functional mapping of stromal signaling microenvironments in the thymus. Annu. Rev. Immunol. 25, 649–679. 10.1146/annurev.immunol.23.021704.115715 (10.1146/annurev.immunol.23.021704.115715) 17291187

[19] Naeher D. Daniels M. Hausmann B. Guillaume P. Luescher I. Palmer E. 2007 A constant affinity threshold for T cell tolerance. J. Exp. Med. 204, 2553–2559. 10.1084/jem.20070254 (10.1084/jem.20070254) 17938233 PMC2118488

[20] Palmer E. Naeher D. 2009 Affinity threshold for thymic selection through a T-cell receptor-co-receptor zipper. Nat. Rev. Immunol. 9, 207–213. 10.1038/nri2469 (10.1038/nri2469) 19151748

[21] Labrecque N. Whitfield L. S. Obst R. Waltzinger C. Benoist C. Mathis D. 2001 How much TCR does a T cell need? Immunity 15, 71–82. 10.1016/S1074-7613(01)00170-4 (10.1016/S1074-7613(01)00170-4) 11485739

[22] Hofmann M. et al. 2004 T cell avidity determines the level of CTL activation. Eur. J. Immunol. 34, 1798–1806. 10.1002/eji.200425088 (10.1002/eji.200425088) 15214028

[23] Corse E. Gottschalk R. Allison J. 2011 Strength of TCR–peptide/MHC interactions and *in vivo* T cell responses. J. Immunol. 186, 5039–5045. 10.4049/jimmunol.1003650 (10.4049/jimmunol.1003650) 21505216

[24] Valitutti S. Müller S. Cella M. Padovan E. Lanzavecchia A. 1995 Serial triggering of many T-cell receptors by a few peptide MHC complexes. Nature 375, 148–151. 10.1038/375148a0 (10.1038/375148a0) 7753171

[25] Valitutti S. Coombs D. Dupré L. 2010 The space and time frames of T cell activation at the immunological synapse. FEBS lett. 584, 4851–4857. 10.1016/j.febslet.2010.10.010 (10.1016/j.febslet.2010.10.010) 20940018

[26] Sykulev Y. Joo M. Vturina I. Tsomides T. Eisen H. 1996 Evidence that a single peptide–MHC complex on a target cell can elicit a cytolytic T cell response. Immunity 4, 565–571. 10.1016/S1074-7613(00)80483-5 (10.1016/S1074-7613(00)80483-5) 8673703

[27] Irvine D. J Purbhoo M. A. Krogsgaard M. Davis M. M 2002 Direct observation of ligand recognition by T cells. Nature 419, 845–849. 10.1038/nature01076 (10.1038/nature01076) 12397360

[28] Huppa J. B. Davis M. M. 2003 T-cell-antigen recognition and the immunological synapse. Nat. Rev. Immunol. 3, 973–983. 10.1038/nri1245 (10.1038/nri1245) 14647479

[29] Rosette C. Werlen G. Daniels M. Holman P. Alam S. Travers P. J. Gascoigne N. R. J. Palmer E. Jameson S. C. 2001 The impact of duration versus extent of TCR occupancy on T cell activation: a revision of the kinetic proofreading model. Immunity 15, 59–70. 10.1016/S1074-7613(01)00173-X (10.1016/S1074-7613(01)00173-X) 11485738

[30] Dushek O. et al. 2011 Antigen potency and maximal efficacy reveal a mechanism of efficient T cell activation. Sci. Signal. 4, ra39 10.1126/scisignal.2001430 (10.1126/scisignal.2001430) PMC4143974 21653229

[31] Das J. Ho M. Zikherman J. Govern C. Yang M. Weiss A. Chakraborty A. K. Roose J. P. 2009 Digital signaling and hysteresis characterize ras activation in lymphoid cells. Cell 136, 337–351. 10.1016/j.cell.2008.11.051 (10.1016/j.cell.2008.11.051) 19167334 PMC2662698

[32] Chakraborty A. Košmrlj A. 2010 Statistical mechanical concepts in immunology. Annu. Rev. Phys. Chem. 61, 283–303. 10.1146/annurev.physchem.59.032607.093537 (10.1146/annurev.physchem.59.032607.093537) 20367082

[33] Chakraborty A. Das J. 2010 Pairing computation with experimentation: a powerful coupling for understanding T cell signalling. Nat. Rev. Immunol. 10, 59–71. 10.1038/nri2688 (10.1038/nri2688) 20029448

[34] Van Den Berg H. Rand D. 2007 Quantitative theories of T-cell responsiveness. Immunol. Rev. 216, 81–92. 10.1111/j.1600-065X.2006.00491.x (10.1111/j.1600-065X.2006.00491.x) 17367336

[35] van der Merwe P. Dushek O 2010 Mechanisms for T cell receptor triggering. Nat. Rev. Immunol. 11, 47–55. 10.1038/nri2887 (10.1038/nri2887) 21127503

[36] McQuarrie D. 1963 Kinetics of small systems. I. J. Chem. Phys. 38, 433–436. 10.1063/1.1733676 (10.1063/1.1733676)

[37] McQuarrie D. Jachimowski C. Russell M. 1964 Kinetics of small systems. II. J. Chem. Phys. 40, 2914–2921. 10.1063/1.1724926 (10.1063/1.1724926)

[38] McQuarrie D. 1967 Stochastic approach to chemical kinetics. J. Appl. Probab. 4, 413–478. 10.2307/3212214 (10.2307/3212214)

[39] Coombs D. Dushek O. Merwe P. 2011 A review of mathematical models for T cell receptor triggering and antigen discrimination. *In* Mathematical models and immune cell biology (eds Molina-París C. Lythe G.), pp. 25–45. Berlin, Germany: Springer.

[40] Stone J. Cochran J. Stern L. 2001 T-cell activation by soluble MHC oligomers can be described by a two-parameter binding model. Biophys. J. 81, 2547–2557. 10.1016/S0006-3495(01)75899-7 (10.1016/S0006-3495(01)75899-7) 11606269 PMC1301723

[41] Stone J. Artyomov M. Chervin A. Chakraborty A. Eisen H. Kranz D. M. 2011 Interaction of streptavidin-based peptide–MHC oligomers (tetramers) with cell-surface TCRs. J. Immunol. 187, 6281–6290. 10.4049/jimmunol.1101734 (10.4049/jimmunol.1101734) 22102724 PMC3237744

[42] Aleksic M. Dushek O. Zhang H. Shenderov E. Chen J. Cerundolo V. Coombs D. Anton van der Merwe P. 2010 Dependence of T cell antigen recognition on T cell receptor–peptide MHC confinement time. Immunity 32, 163–174. 10.1016/j.immuni.2009.11.013 (10.1016/j.immuni.2009.11.013) 20137987 PMC2862301

[43] Govern C. Paczosa M. Chakraborty A. Huseby E. 2010 Fast on-rates allow short dwell time ligands to activate T cells. Proc. Natl Acad. Sci. 107, 8724–8729. 10.1073/pnas.1000966107 (10.1073/pnas.1000966107) 20421471 PMC2889346

[44] Lauffenburger D. Linderman J. 1996 Receptors: models for binding, trafficking, and signaling. Oxford, UK: Oxford University Press.

[45] Drews T. Katsoulakis M. Tsapatsis M. 2005 A mathematical model for crystal growth by aggregation of precursor metastable nanoparticles. J. Phys. Chem. B 109, 23879–23887.(10.1021/jp0537299) 16375373

[46] Svetic R. MacCluer C. Buckley C. Smythe K. Jackson J. 2004 A metabolic force for gene clustering. Bull. Math. Biol. 66, 559–581. 10.1016/j.bulm.2003.09.008 (10.1016/j.bulm.2003.09.008) 15006449

[47] Kierzek A. 2002 STOCKS: STOChastic Kinetic Simulations of biochemical systems with Gillespie algorithm. Bioinformatics 18, 470–481. 10.1093/bioinformatics/18.3.470 (10.1093/bioinformatics/18.3.470) 11934747

[48] Yang J. Hlavacek W. 2011 Scaffold-mediated nucleation of protein signaling complexes: elementary principles. Math. Biosci. 232, 164–173. 10.1016/j.mbs.2011.06.003 (10.1016/j.mbs.2011.06.003) 21683720 PMC3137898

[49] Gardiner C. 1985 Stochastic methods. Berlin, Germany: Springer.

[50] van Kampen N 2007 Stochastic processes in physics and chemistry. Amsterdam, The Netherlands: North-Holland.

[51] Gillespie D. 1992 Markov processes: an introduction for physical scientists. New York, NY: Academic Press.

[52] Altan-Bonnet G. Germain R. 2005 Modeling T cell antigen discrimination based on feedback control of digital ERK responses. PLoS Biol. 3, e356 10.1371/journal.pbio.0030356 (10.1371/journal.pbio.0030356) 16231973 PMC1262625

[53] Lipniacki T. Hat B. Faeder J. R. Hlavacek W. S. 2008 Stochastic effects and bistability in T cell receptor signaling. J. Theor. Biol. 254, 110–122. 10.1016/j.jtbi.2008.05.001 (10.1016/j.jtbi.2008.05.001) 18556025 PMC2577002

[54] Grimmett G. Stirzaker D. 2001 Probability and random processes. Oxford, UK: Oxford University Press.

[55] Huppa J. Axmann M. Mörtelmaier M. Lillemeier B. Newell E. Brameshuber M. Klein L. O. Schüz G. J. Davis M. M. 2010 TCR–peptide–MHC interactions in situ show accelerated kinetics and increased affinity. Nature 463, 963–967. 10.1038/nature08746 (10.1038/nature08746) 20164930 PMC3273423

[56] Huang J. Zarnitsyna V. Liu B. Edwards L. Jiang N. Evavold B. D. Zhu C. 2010 The kinetics of two dimensional TCR and pMHC interactions determine T cell responsiveness. Nature 464, 932–936. 10.1038/nature08944 (10.1038/nature08944) 20357766 PMC2925443

[57] Redner S. 2001 A guide to first-passage processes. Cambridge, UK: Cambridge University Press.

[58] Taylor H. Karlin S. 1998 An introduction to stochastic modeling. New York, NY: Academic Press.

[59] Zarnitsyna V. Huang J. Zhang F. Chien Y. Leckband D. Zhu C. 2007 Memory in receptor–ligand-mediated cell adhesion. Proc. Natl Acad. Sci. USA 104, 18037–18042.(10.1073/pnas.0704811104) 17991779 PMC2084292

[60] Robert P. Aleksic M. Dushek O. Cerundolo V. Bongrand P. van der Merwe P. A. 2012 Kinetics and mechanics of two-dimensional interactions between T cell receptors and different activating ligands. Biophys. J. 102, 248–257. 10.1016/j.bpj.2011.11.4018 (10.1016/j.bpj.2011.11.4018) 22339861 PMC3260781

[61] Mckeithan T. 1995 Kinetic proofreading in T-cell receptor signal transduction. Proc. Natl Acad. Sci USA 92, 5042–5046. 10.1073/pnas.92.11.5042 (10.1073/pnas.92.11.5042) 7761445 PMC41844

[62] Salazar C. Höfer T. 2009 Multisite protein phosphorylation–from molecular mechanisms to kinetic models. FEBS J. 276, 3177–3198. 10.1111/j.1742-4658.2009.07027.x (10.1111/j.1742-4658.2009.07027.x) 19438722

[63] Dushek O. Das R. Coombs D. 2009 A role for rebinding in rapid and reliable T cell responses to antigen. PLoS Comput. Biol. 5, e1000578 10.1371/journal.pcbi.1000578 (10.1371/journal.pcbi.1000578) 19956745 PMC2775163

[64] Gascoigne N. Zal T. Alam S. 2001 T-cell receptor binding kinetics in T-cell development and activation. Expert Rev. Mol. Med. 2, 1–17. 10.1017/S1462399401002502 14987373

[65] Werlen G. Hausmann B. Naeher D. Palmer E. 2003 Signaling life and death in the thymus: timing is everything. Science 299, 1859–1863. 10.1126/science.1067833 (10.1126/science.1067833) 12649474

[66] Bevan M. 1997 In thymic selection, peptide diversity minireview gives and takes away. Immunity 7, 175–178. 10.1016/S1074-7613(00)80520-8 (10.1016/S1074-7613(00)80520-8) 9285402

[67] Zinkernagel R. Ehl S. Aichele P. Oehen S. Kündig T. Hengartner H. 1997 Antigen localisation regulates immune responses in a dose-and time-dependent fashion: a geographical view of immune reactivity. Immunol. Rev. 156, 199–209. 10.1111/j.1600-065X.1997.tb00969.x (10.1111/j.1600-065X.1997.tb00969.x) 9176709

[68] Wedagedera J. Burroughs N. 2006 T-cell activation: A queuing theory analysis at low agonist density. Biophys. J. 91, 1604–1618. 10.1529/biophysj.105.066001 (10.1529/biophysj.105.066001) 16766611 PMC1544309

[69] Dushek O. Coombs D. 2008 Analysis of serial engagement and peptide-MHC transport in T cell receptor microclusters. Biophys. J. 94, 3447–3460. 10.1529/biophysj.107.116897 (10.1529/biophysj.107.116897) 18227132 PMC2292378

[70] Allen L. 2003 An introduction to stochastic processes with applications to biology. Englewood Cliffs, NJ: Prentice Hall.

[71] Gillespie D. 1977 Exact stochastic simulation of coupled chemical reactions. J. Phys. Chem. 81, 2340–2361. 10.1021/j100540a008 (10.1021/j100540a008)

[72] Gillespie D. 1992 A rigorous derivation of the chemical master equation. Physica A 188, 404–425. 10.1016/0378-4371(92)90283-V (10.1016/0378-4371(92)90283-V)

